# NIR-laser-triggered gadolinium-doped carbon dots for magnetic resonance imaging, drug delivery and combined photothermal chemotherapy for triple negative breast cancer

**DOI:** 10.1186/s12951-021-00811-w

**Published:** 2021-03-02

**Authors:** Qunjiao Jiang, Li Liu, Qiuying Li, Yi Cao, Dong Chen, Qishi Du, Xiaobo Yang, Dongping Huang, Renjun Pei, Xing Chen, Gang Huang

**Affiliations:** 1 School of Public Health, Guangxi Medical University, Nanning, 530000 China; 2 State Key Laboratory of Non-Food Biomass and Enzyme Technology, Guangxi Academy of Sciences, Nanning, 530007 China; 3 Key Laboratory of Nano-Bio Interface, Suzhou Institute of Nano-Tech and Nano-Bionics, Chinese Academy of Sciences, Suzhou, 215123 China

**Keywords:** Carbon dots, Magnetic resonance imaging, Photothermal chemotherapy, Drug delivery, Triple negative breast cancer therapy

## Abstract

**Background:**

Owing to high genetic diversities of tumor cells and low response rate of standard chemotherapy, patients with triple negative breast cancer (TNBC) have short progression-free survivals and poor outcomes, which need to explore an effective approach to improve therapeutic efficacy.

**Methods:**

Novel gadolinium doped carbon dots (Gd@CDs) have been designed and prepared through hydrothermal method with 3,4-dihydroxyhydrocinnamic acid, 2,2′-(ethylenedioxy)bis(ethylamine) and gadolinium chloride. The synthesized nanostructures were characterized. Taking advantage of good biocompatibility of Gd@CDs, a nanoplatform based on Gd@CDs has been developed to co-deliver chemotherapy drug doxorubicin hydrochloride (Dox) and a near-infrared (NIR) photothermal agent, IR825 for magnetic resonance imaging (MRI) guided photothermal chemotherapy for TNBC.

**Results:**

The as-synthesized Dox@IR825@Gd@CDs displayed favorable MRI ability in vivo*.* Upon NIR laser irradiation, Dox@IR825@Gd@CDs could convert the NIR light to heat and efficiently inhibit tumor growth through photothermal chemotherapy in vitro and in vivo. Additionally, the impact of photothermal chemotherapy on the murine motor coordination was assessed by rotarod test. Dox@IR825@Gd@CDs presented low toxicity and high photothermal chemotherapy efficiency.

**Conclusion:**

A noble theranostic nanoplatform (Dox@IR825@Gd@CDs) was developed that could be tailored to achieve loading of Dox and IR825, intracellular delivery, favorable MRI, excellent combination therapy with photothermal therapy and chemotherapy to enhance therapeutic effect against TNBC cells. This study will provide a promising strategy for the development of Gd-based nanomaterials for MRI and combinational therapy for TNBC.

**Graphic abstract:**

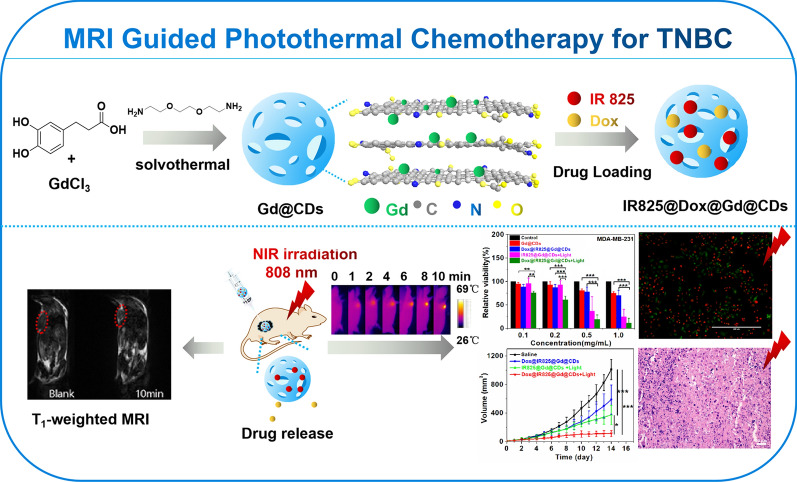

## Background

The imaging-guided theranostics for the diagnosis and treatment of cancer, which seeks to address traditional medical challenges including poor bioavailability, systemic toxicity and low efficiency, now encompasses wider areas of cancer therapy [1–3]. An ideal therapeutic platform should possess the capability to locate tumors, achieve deep penetration, guide cancer therapy and improve therapeutic effect [1, 2]. Magnetic resonance imaging (MRI) is regarded as one of most powerful non-invasive techniques for true 3D and high quality multiplanar image of soft tissue in vivo [4, 5]. Gadolinium (Gd), possessing unpaired electrons in its outermost shell, not only creates strong local proton environment but also contributes to the overall signal intensity of T_1_-weighted images [6]. Although Gd^3+^-based contrast probes, such as Gd-DTPA and Gd-DOTA, have play a crucial part in clinical MRI, the sensitivity of these contrast agents for distinguishing the tumors from the other surrounding organs still remains to be enhanced [5]. Moreover, since free Gd^3+^ ions are toxic in vivo, the potential Gd retention within the skin, bones, and solid organs may cause a high toxicity profile by calcium antagonist, nephrogenic systemic fibrosis (NSF) in patients with impaired renal function [7–10]. To address these questions, new generation of Gd-containing nanoprobes, including Gd_2_O_3_ nanoparticles, Gd-loaded silica nanoparticles, Gd-doped Fe_3_O_4_ nanoparticles and Gd-encapsulated carbon dots (CDs), have been developed to suppress the side-effect caused by the Gd leakage [11]. Among these nanoparticles, CDs have shown remarkable physicochemical and biological properties, such as simple preparation, unique nanostructures, ease of functionalization, good photostability, low toxicity, excellent biocompatibility and cell membrane permeability [12, 13]. Therefore, Gd-encapsulated CDs have been recognized as smart candidates as MRI probes and drug carriers for cancer therapy.

Triple negative breast cancer (TNBC) lacks estrogen receptor, progesterone receptor and human epidermal growth factor receptor [14], leading to high heterogeneity, extremely aggressive tumor biology and a low prognosis [15]. Although some TNBC patients are sensitive to anthracyclines and taxanes, long-term chemotherapy evolves drug resistance, resulting in poor overall survivals [16, 17]. To deal with these drawbacks, synergistic approach involving two or more therapeutic modalities can enhance therapeutic efficacy, because single chemotherapy could not get rid of all the cancer cells, and might increase the risk of the recurrence or metastasis [18–21]. In addition to conventional chemotherapy, photothermal therapy (PTT), as a minimally invasive and highly effective cancer treatment method, could effectively convert the photo energy into heat to kill cancer cells under light irradiation, increase cell membrane permeability to improve the uptake of drug, and further enhance the effectiveness of chemotherapy [21–23]. Photothermal chemotherapy could be recognized as one of the most effective combinatorial treatments to synergistically destroy tumors while preventing recurrence and metastasis [24–26]. Hence, an ideal therapeutic platform combined photothermal chemotherapy need to explore to improve therapeutic efficacy against TNBC.

**Scheme 1:**
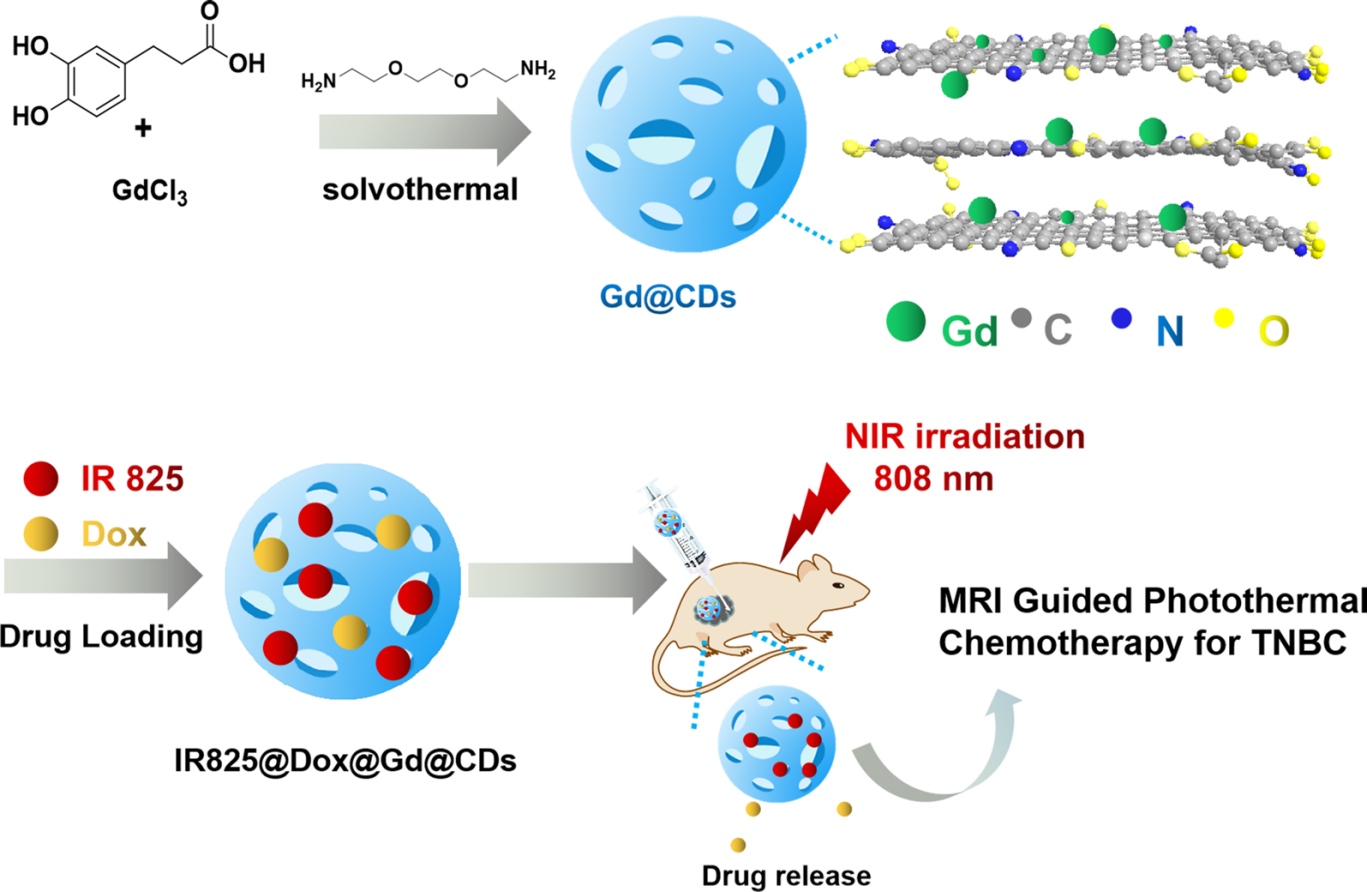
Design strategy of a multifunctional carbon nanoplatform based on Gd@CDs to realize MRI guided photothermal chemotherapy for TNBC

In this work, Gd-encapsulated carbon dots (Gd@CDs) were synthesized through a hydrothermal method with 3,4-dihydroxyhydrocinnamic acid, 2,2′-(ethylenedioxy)bis(ethylamine) and gadolinium chloride. A nanoplatform Dox@IR825@Gd@CDs has been developed by delivering chemotherapy drug Dox and photothermal agent IR825 for MRI guided combination photothermal chemotherapy for TNBC (Scheme 1). This study will provide a promising strategy for the development of Gd-based nanomaterials for MRI and combinational therapy of TNBC.

## Materials and methods

### Materials

3,4-dihydroxyhydrocinnamic acid (DHCA), 2,2′-(ethylenedioxy)bis(ethylamine) (EDA), anhydrous gadolinium chloride (GdCl_3_), doxorubicin hydrochloride (Dox), 1,1-diphenyl-2-picrylhydrazyl (DPPH) and other chemical reagents were bought from Energy Chemical Co. Ltd without further purification. Fetal bovine serum (FBS), trypsin, penicillin and streptomycin were obtained from Biological Industries (Kibbutz Beit Haemek, Israel). RPMI Medium 1640 basic (1×) was purchased from Thermo Fisher Biochemical Products co. LTD (Beijing, China). Cell Counting Kit-8 (CCK-8) was bought from Dojindo Chemical technology co. LTD (Shanghai, China). The murine breast cancer 4T1 cell line and human breast cancer MDA-MB-231 cell line were obtained from Cell Bank of Chinese Academy of Sciences.

### Preparation of Gd@CDs

369.5 mg of DHCA, 0.584 mL of EDA, 78 mg of GdCl_3_ and 20 mL of deionized water were added into an autoclave and heated to 200 ℃ for 5 h. After cooling to room temperature, the mixture was centrifuged at 6000 rpm for 10 min to remove brown particles. The supernatant solution was filtered three times through a 0.22 µm membrane to remove large particles, and then transferred into a dialysis membrane (MWCO = 500) for dialysis against deionized water for 72 h. Gd@CDs were collected and lyophilized for further characterization.

### Characterization of Gd@CDs

Nuclear magnetic resonance (NMR) spectra were recorded in D_2_O on Agilent Technologies 800/54 premium. Fourier transform infrared spectroscopy (FT-IR) was performed on Thermo Scientific Nicolet iS10 spectrometer. UV–vis absorption spectra were collected on Agilent Cary 60 spectrophotometer and Shimadzu UV-1900 spectrophotometer. The fluorescence spectra were measured on Hitachi F-7000. The fluorescence lifetime and quantum yield were recorded on an Edinburgh Instruments FLS980. Gadolinium in Gd@CDs was quantified by Thermo Fisher iCAP Qnova Series inductively coupled plasma mass spectrometry (ICP-MS). X-ray photoelectron spectroscopy (XPS) analysis was recorded by Kratos Axis Utra Dld. Raman spectra were measured on Labram Aramis with an excitation wavelength of 532 nm. The morphologies were observed with FEI Tecnai G2 F30 S-TWIN TEM. The hydrodynamic diameter and zeta potential of nanoparticles were measured by a PSS-Nicomp 380ZLS.

### Biocompability of Gd@CDs

Cellular viability assays. 293 T cells were incubated with standard DMEM culture medium with 10% FBS and 1% penicillin/streptomycin at 37 °C under 5% CO_2_ atmosphere. 293 T cells (1 × 10^4^ cells per well) were seeded onto 96-well plate until adherent and then treated with different concentrations of Gd@CDs (0.1, 0.2, 0.5, 1.0 mg/mL), while PBS was used as control. After then, 293 T cells were incubated for 24 h. The absorbance at 450 nm was recorded by Thermo Fisher multiskan Go spectrophotometer. Then, the viability of cells was calculated according to Eq. (1):

1 $$\mathrm{Viability }\left(\mathrm{\%}\right)=\frac{{A}_{s}-{A}_{b}}{{A}_{c}-{A}_{b}}\times 100$$

A_s_, A_b_, A_c_ were the absorbance of sample, blank and control groups, respectively. Three replicates were carried out.

Toxicity study in vivo. Kunming mice (n = 18, female, 5 weeks) were purchased from Guangxi Medical University Laboratory Animal Center and used under protocols approved by Guangxi Medical University Laboratory Animal Center. Kunming mice were randomly divided into 3 groups. The mice were injected via tail vein every three days (0, 3, 6, 9, 12, 15 Day) and treated as follows: (a) saline (100 μL), (b) Gd@CDs (0.3 mg/kg), (c) Gd@CDs (3 mg/kg). The body weight changes of the injected mice were monitored. The mice were sacrificed at 16 d to collect blood for blood biochemical assay. In addition, the heart, liver, spleen, lung and kidney of mice were gathered and weighted. All tissues were stained with *H&E* staining. However, the Balb/c mice were sacrificed after 24 h post injection for histopathology examination: (a) saline, (b) Gd@CDs (0.1 mg/kg), (c) Gd@CDs (1 mg/kg), (d) Gd@CDs (10 mg/kg).

### Synthesis of Dox@IR825@Gd@CDs

Gd@CDs (40 mg) were dispersed in deionized water. Then 0.2 mL of DMSO solution containing 4 mg of Dox and 4 mg of IR825 was dropwise added. The mixture was incubated at 37℃ on a shaker for 24 h, yielding Dox@IR825@Gd@CDs solution, which was purified by dialysis against water for 24 h before lyophilization. Drug loading efficiency and encapsulation were calculated according to Eqs. (2), (3).

2 $$\text{Drug loading efficiency }(\mathrm{\%})= \frac{mass\; of\; the\; encapsulated\; drug}{total\; mass \; of\; the\; sample}\times 100$$

3 $$\text{Drug encapsulation efficiency }(\mathrm{\%})= \frac{mass\; of\; the\; encapsulated\; drug}{ mass\; of\; the\; initial\; drug}\times 100$$

The hydrodynamic diameters of Dox@IR825@Gd@CDs in the presence of PBS with 10% FBS were recorded on a PSS-Nicomp 380ZLS during 99 h.

### *Drug release *in vitro

Dox@IR825@Gd@CDs dialysate (2 mL) was transferred into new dialysis membrane (MWCO = 3500 D) and immersed in 25 mL of PBS buffer at pH 4.92 or 7.38 for 120 h. 4 mL of medium outside the dialysis membrane was taken out and an equal volume of fresh PBS was replenished at desired time points. The release behaviors of Dox and IR825 were investigated using a dialysis membrane and the absorbance was measured at 530 nm and 790 nm, respectively. Three replicates were carried out.

### Photothermal effect evaluations

Dox@IR825@Gd@CDs solutions (0.1, 0.2, 0.5, 1.0 mg/mL) were exposed to an 808 nm laser irradiation (3.0 W) for 5 min. Solution temperatures were recorded by thermometer and thermal imaging system (Fluke Ti450) at an interval time to evaluate the photothermal property of Dox@IR825@Gd@CDs. Three replicates were carried out. Additionally, to evaluate photothermal stability, Dox@IR825@Gd@CDs (1 mg/mL) solution was irradiated with an 808 nm laser (3.0 W) for 5 min and then naturally cooled down to room temperature. The temperature changes were monitored with a time intervals of 1 min during 5 cycles of laser on / off irradiation.

### Cellular uptake

4T1 cells were incubated with standard RPMI-1640 culture medium with 10% FBS and 1% penicillin/streptomycin at 37 °C under 5% CO_2_ atmosphere. 4T1 cells were seeded on glass coverslips (10^5^ cells per slip) until adherent and incubated with Dox@IR825@Gd@CDs (0.5 mg/mL) for 4 h. After being washed three times with PBS to remove the excess nanoparticles, the cells were fixed by 4% paraformaldehyde and washed again with PBS. The cells were washed with PBS containing 0.2% Triton-100 for easy permeability. Afterwards, the cells were stained with DAPI and imaged by EVOS FL Auto. Three replicates were carried out.

### *Combined photothermal chemotherapy *in vitro

The cellular viability was assessed against 4T1 cells and MDA-MB-231 cells by a CCK-8 assay. MDA-MB-231 cells were incubated with standard RPMI-1640 culture medium with 10% FBS and 1% penicillin/streptomycin at 37 °C under 5% CO_2_ atmosphere. 4T1 cells (5 × 10^3^ cells per well) and MDA-MB-231 cells (1 × 10^4^ cells per well) were seeded onto 96-well plate until adherent and then treated with different concentrations of Gd@CDs, IR825@Gd@CDs and Dox@IR825@Gd@CDs (0.1, 0.2, 0.5, 1.0 mg/mL), while PBS was used as control. IR825@Gd@CDs groups were irradiated with a laser (808 nm, 3 W) for 5 min. Dox@IR825@Gd@CDs groups were treated in the absence or presence of a laser irradiation (808 nm, 3 W) for 5 min. After then, the cells were further incubated for 12 h. The viability was examined by CCK-8 assay. The absorbance at 450 nm was recorded by Thermo Fisher multiskan Go spectrophotometer. The viability of cells was calculated according to Eq. (1). Three replicates were carried out.

Calcein-AM/PI assay against 4T1 cells and MDA-MB-231 cells were also performed to evaluate the antitumor efficiency in vitro. Cells (5 × 10^4^ cells per well) were seeded onto 24-well plate and treated by incubation of different formulations (Gd@CDs, IR825@Gd@CDs, Dox@IR825@Gd@CDs, 0.5 mg/mL) with/without irradiation in a similar way as the cellular viability assays, described above. The resultant cells were co-stained with calcein-AM and PI, and imaged by EVOS FL Auto. Three replicates were carried out.

### MRI in vitro and in vivo

MRI in vitro was acquired from a 1.5 T Philip MRI instrument under following parameters: TR/TE = 3500/20 ms, NSA = 1, FOV = 60 × 60 mm^2^, matrix size = 256 × 256, section thickness = 1 mm. A series of diluted Gd@CDs solution were imaged comparing to commercial contrast agent gadopentetate dimeglumine (Gd-DTPA) with the same concentrations of Gd.

MRI in vivo was collected using a 1.5 T scanner (HT-MRSI60-35A, Shanghai Huantong Science and Education Equipment Co., Ltd). The parameters for T1 imaging were set as follows: TR/TE = 100 /14.12 ms, matrix = 512 × 256, FOV = 80 × 45 mm^2^, slice thickness = 0.3 mm, 128 slices and no gap between slices. Female BALB/c mice (4 weeks old) were obtained from Changzhou Cavens Laboratory Animal Co., Ltd. and acclimated for 2 weeks before use. To fabricate a xenograft tumor model, the armpit of mice was subcutaneously injected with 2 × 10^6^ 4T1 cells suspended in 100 μL of PBS. When the tumor size reached about 10 mm in diameter after about 2 weeks, the mice were used for MRI experiments. The tumor-bearing mice were injected intravenously (n = 2) or in situ (n = 2) with Dox@IR825@Gd@CDs. MR images were acquired at 0, 1, 2, 3, 4, 5, 6 and 12 h after intravenous injection and at 10 min after in situ injection.

### Combined photothermal chemotherapy in vivo

Animal Model: nude mice (≈ 16 g, female, 5 weeks) were purchased from Guangxi Medical University Laboratory Animal Center and were randomly divided into 4 groups. For the 4T1 murine breast tumor model, 4T1 cells (4 × 10^5^) were subcutaneously injected. The mice were used when their tumor volumes approached 10 mm^3^. The tumor-bearing mice were injected the sample solution (20 µL, 8 mg) in situ and treated as follows: (a) saline (n = 5), (b) Dox@IR825@Gd@CDs (n = 5), (c) IR825@Gd@CDs (808 nm laser, 1.5 W, 10 min, n = 5), (d) Dox@IR825@Gd@CDs (808 nm laser, 1.5 W, 10 min, n = 5). The thermal images of the mice were observed by Fluke thermal imaging after treatment. Tumor size and body weight were also recorded. Tumor volume (*V*) was calculated by the following equation: *V* = *ab*^2^/2 (*a* and *b* denoted the length and width of tumor). The heart, liver, spleen, lung, kidney and tumor of mice were gathered and weighted after 14 days treatments. All tissues were stained with *H&E* staining.

### Biodistribution of Dox@IR825@Gd@CDs

To evaluate the biodistribution of Dox@IR825@Gd@CDs in vivo, the tumor-bearing mice were sacrificed after 24 h post-treatment (n = 4). The heart, liver, spleen, lung, kidney and tumor were weighted and predigested with 5 mL of HNO_3_ by microwave digestion (CEM MARS6 CLASSIC). The gadolinium contents were measured by ICP-MS.

### Rotarod test

To evaluate the impact of combination therapy on murine motor coordination, the nude mice, tumor-bearing mice before and after 14 days treatments were assessd by rotarod test. The initial roating speed was set at 5 rpm for 10 s, then uniformly increased to 25 rpm within 1 min. The duration was automatically recorded by Ugo Basile 47,650 RotaRod.

### Statistical analysis

The data were expressed as mean ± standard deviation (SD). Results of biochemistryanalysis, mouse weight, tumor weight, temperature increments of various amount of Dox@IR825@Gd@CDs suspension under NIR laser irradiation, tumor volume assays and rotarod test were performed using multiple comparisons in analysis of variance (ANOVA). Comparison of the release rate of Dox and IR825 in different pH solutions and temperature increments of Dox@IR825@Gd@CDs suspension under NIR laser irradiation for five cycles have been made with t test. *P* values less than 0.05 were defined as statistically significant.

### Live subject statement

All experiments were approved by the Ethics Committee of Guangxi Medical University and carried on in strict compliance with the relevant laws and institutional guidelines of Guangxi Medical University, Nanning, China (20140307A, 20140307B).

## Results and discussion

### Preparation and characterization of Gd@CDs

Gd@CDs were prepared via the typical solvent-thermal method, in which DHCA and anhydrous GdCl_3_ were used as the carbon source and MRI contrast agent, while EDA was as a passivation agent. Gd@CDs were purified by filtration and dialysis to remove nanoparticles aggregations and unreacted precursors. Then, Gd@CDs were further characterized.

**Fig. 1:**
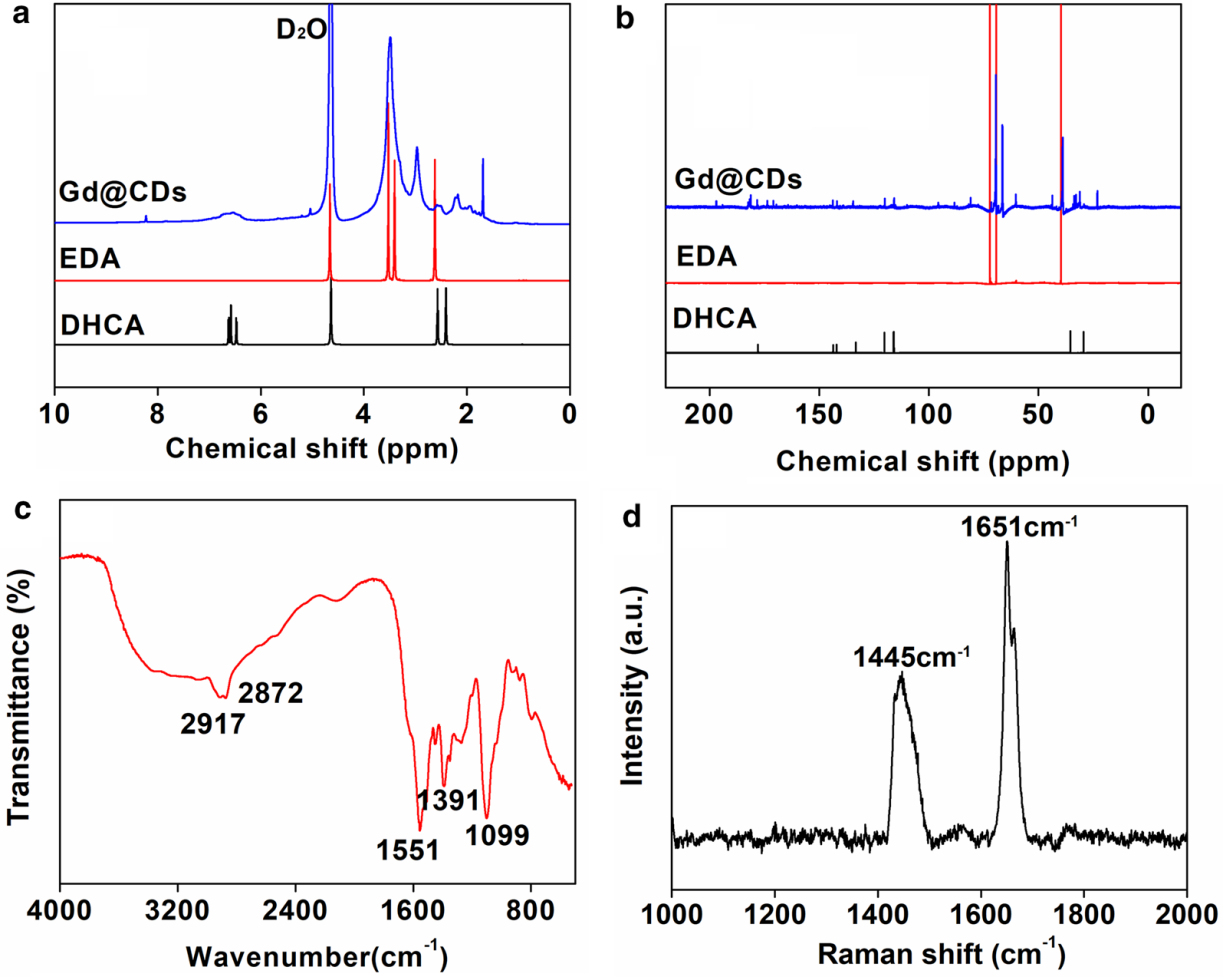
**a** ^1^HNMR spectrum, **b** ^13^CNMR spectrum, **c** Fourier-infrared spectrum and **d** Raman spectrum of Gd@CDs

Comparing with the ^1^H NMR spectra of EDA and DHCA, the chemical shifts of Gd@CDs at 1.39–4.39 ppm were assigned to the protons on saturated carbons and the appearing signals in the range of 6.24 and 7.03 ppm were attributed to the protons on the amine groups (Fig. 1a). The chemical shifts at 8.23 ppm revealed the protons on the aromatic ring. In the ^13^C NMR spectrum of Gd@CDs (Fig. 1b), three sharp characteristic peaks appeared in the chemical shifts of 38.95, 66.42 and 69.40 ppm, which were attributed to the carbon atoms: –O–CH_2_*C*H_2_–NH_2_, –O–CH_2_*C*H_2_–O–, and –O–*C*H_2_CH_2_–NH_2_. Other weak characteristic peaks were roughly divided into two regions: the saturated carbon atoms in the region of 22.9–69.34 ppm, and the unsaturated carbon atoms region (C=C), aromatic with carbon–carbon double bond at 115.94–143.89 ppm and carbon–oxygen double bond at 170.64–196.84 ppm [27].

**Fig. 2:**
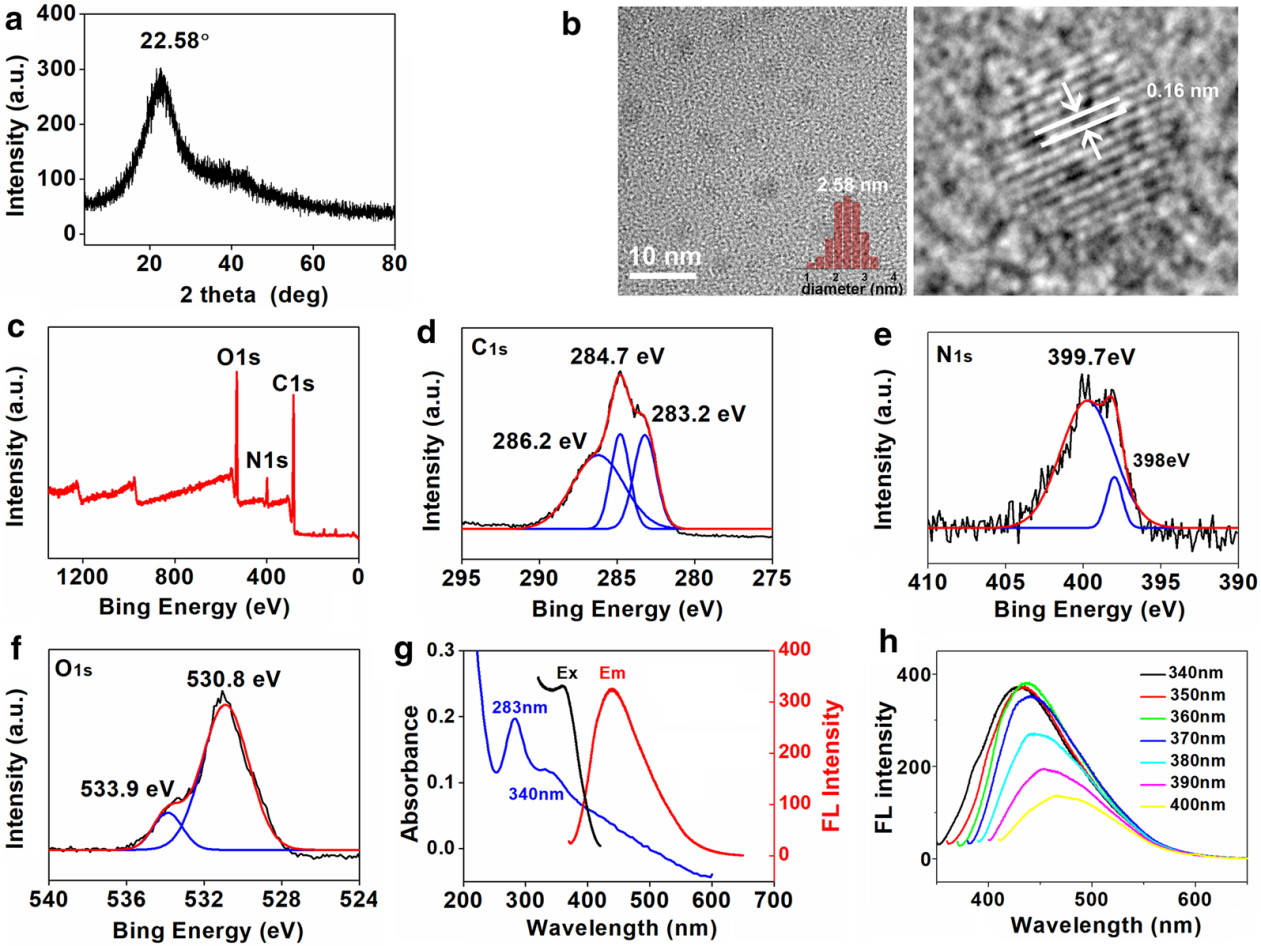
Characterizations of Gd@CDs. **a** XRD spectrum; **b** HRTEM imaging (inset, statistical analysis by nanomeasurer software); **c** Scanned XPS spectrum, **d** C_1s_ XPS spectrum; **e** N_1s_ XPS spectrum; **f** O_1s_ XPS spectrum; **g** The absorbance, excitation and emission spectra; **h** The emission spectra under various excitation wavelengths

In FT-IR spectra (Fig. 1c), a very broad peak from 3700 to 3000 cm^−1^ indicated the presence of unsaturation hydrogen (=C–H) stretches and exchangeable protons, typically from hydroxyl groups, amide groups or carboxylic acid groups [28]. The peaks of 2917 and 2872 cm^−1^ were attributed to C–H stretches of alkyl groups [28]. The signals at 1551 cm^−1^ belonged to alkene stretches [29, 30]. The intense IR absorptions at 1391 cm^−1^ and 1099 cm^−1^ were attributed to C–O and C–N stretches (Fig. 1c) [29, 30]. As shown in Raman spectrum (Fig. 1d), the peaks of 1445 cm^−1^ and 1651 cm^−1^ belonged to D band (sp^3^ hybridization) and G band (sp^2^ hybridization), respectively [31]. The integrated intensity ratio of D and G bands (*I*_D_/*I*_G_ = 0.54) was directly proportional to the surface defect density of carbon points, indicating a high degree of graphitization on the surface of Gd@CDs [30, 32]. For the XRD pattern (Fig. 2a), a weak diffraction peak at 22.58° was due to the amorphous carbon structure, similar to graphene lamellar structure [30, 33, 34]. The morphologies of Gd@CDs were observed by the HRTEM images (Fig. 2b), exhibiting the spherical shape with an average diameter of 2.58 nm and a lattice fringe distance of 0.16 nm. Gd@CDs exhibited a zeta potential of -16.31 mV and a size of 308.8 nm by dynamic light scattering (DLS) (Additional file 1: Figure S1a). However, the size of Gd@CDs by the HRTEM images was much smaller than that of DLS result. The dry particles were observed in HRTEM image, while the DLS measured the hydrodynamic size of nanoparticles in suspension, both core and surrounding solvent layer. Furthermore, it was inevitable for Gd@CDs to aggregate due to low surface charge and hydrogen bonds formation between –NH_2_ and –COOH which Gd@CDs were functionalized with. Hence, the DLS results exhibited larger size than that of HRTEM images.

Gd@CDs contained C, O and N elements (Fig. 2c). The deconvoluted C1s showed three peaks (Fig. 2d), including C=C at 283.2 eV, C–C/C–N at 284.7 eV and C–O/C=O/C = N at 286.2 eV [35]. In N_1s_ spectra (Fig. 2e), the signals at 398 and 399.7 eV were attributed to C–N/N–C=O and -NH_2_ [36]. The fitted O_1s_ spectra gave two peaks (Fig. 2f). Peaks at 530.8 and 533.9 eV were consistent to C=O and C-O, demonstrating that Gd@CDs contained -OH and -COOH groups [29, 37]. The concentration of Gd quantitated by ICP-MS was 0.459% in Gd@CDs.

Gd@CDs showed a broad absorption band between 200 to 500 nm, with two shoulders appearing at 283 nm and 340 nm (Fig. 2g). The characteristic absorption peak at 283 nm was attributed to the absorption peak position of the π-π* transition of C = C skeleton on the aromatic ring. The absorption peak at 340 nm belonged to the n-π* transition of C = O in the carboxyl group [29, 38]. The excitation-dependent fluorescence properties of Gd@CDs were also recorded (Fig. 2h). When the excitation wavelength was set at 360 nm, Gd@CDs revealed a maximum emission peak at 437 nm. Such wavelength-dependent fluorescence was also similar to conventional C-dots [39]. The fluorescence lifetime of Gd@CDs was 3.58 ns and the quantum yield was 26.84% (Additional file 1: Figure S1b). The fluorescence properties kept constant at different temperatures (Additional file 1: Figure S1c) and various NaCl concentrations (Additional file 1: Figure S2a), and in the presence of amino acids (Additional file 1: Figure S2b). The fluorescence intensity of Gd@CDs slightly decreased at acid condition (pH < 7) or base condition (pH > 7, Additional file 1: Figure S2c). The maximum fluorescent quenching of Gd@CDs was occurred in presence of Fe^3+^ and Cu^2+^ (Additional file 1: Figure S2d), which could be caused by electron transfer from the negative Gd@CDs to metal ions [36, 40]. Gd@CDs held great photostability and resistance to photo bleaching upon Xe lamp illumination (Additional file 1: Figure S3a). The radical scavenging activity of Gd@CDs was assessed by DPPH method, showing that the ratio of quenching DPPH radicals improved with the increase of the concentration of Gd@CDs and the EC_50_ was 6.54 μg/mL, which illustrated Gd@CDs were susceptible to nitrogen radicals (Additional file 1: Figure S3b).

Hemolysis assay was examined to explore the blood compatibility of Gd@CDs. No significant hemolysis phenomenon was found in different concentrations of Gd@CDs solution, indicating Gd@CDs caused little damage to red blood cells and were biocompatible with blood (Additional file 1: Figure S4a). To explore the cytotoxicity of Gd@CDs, we first investigated Gd@CDs against human embryonic kidneys cells (293 T cells) by CCK-8 assay (Additional file 1: Figure S4b). The cell viability was still above 90% even when the concentration of Gd@CDs increased to 1 mg/mL after 24 h incubation. The results indicated the lower cytotoxicity of Gd@CDs in vitro.

In vivo histological toxicity analysis was conducted to assess cell damages of the major organs including the heart, liver, spleen, lung and kidney after 24 h post injection of Gd@CDs solution (Additional file 1: Figure S5). Comparing with the control groups, there were no obvious damages in the Gd@CDs treated groups, such as inflammatory response, pulmonary fibrosis, necrosis or damages in major organs.

Long term toxicity of Gd@CDs in vivo was also conducted on healthy Kunming mice model for 16 days. The mice were injected saline or Gd@CDs solution (0.3 mg/kg and 3 mg/kg) via tail vein. The body weights of the mice were monitored during the test. There were no statistical differences in body weights at 0 d and 15 d compared with the control group (Additional file 1: Figure S6). At 16 d, blood was collected for serum biochemistry assay. Total protein (TP), alanine aminotransferase (ALT), aspartate aminotransferase (AST), alkaline phosphatase (ALP), blood urea nitrogen (BUN), total cholesterol (TC), and triglyceride (TG) were evaluated, which reflect liver and kidney functions. Compared with the normal saline treatment group, there were no differences in the values of biochemical markers of Gd@CDs treated groups (Table 1), suggesting no obvious hepatic or kidney disorder of mice induced by Gd@CDs treatment. From the histopathology of treated mice tissues, there were almost no apparent histopathological abnormalities or lesions observed in the heart, kidney, liver, and spleen, compared with the control groups (Additional file 1: Figure S7). However, the H&E staining images of the lung tissues revealed the presence of peribronchial and perivascular cellular infiltrates at 0.3 mg/kg and 3 mg/kg dosages, which indicated that Gd@CDs evoked moderate lung inflammatory responses (Additional file 1: Figure S7). Similar phenomena were also observed in the other carbonaceous nanomaterials, such as polyamine-based CDs [41], carboxylated CDs [42] and carbon nanotubes [43, 44]. Since the lung hosted multiple populations of macrophages, Gd@CDs may alter the steady state of oxidative stress induced by excessive generation of reactive oxygen species and depletion of antioxidants levels, causing production of proinflammatory cytokines in lung cells [41, 45, 46]. Necrosis was not found in histological samples. Above all, Gd@CDs exhibited good biocompatibility in vivo. Therefore, Gd@CDs were suitable for further application in biomaterials.

### Preparation and the properties of Dox@IR825@Gd@CDs

**Fig. 3:**
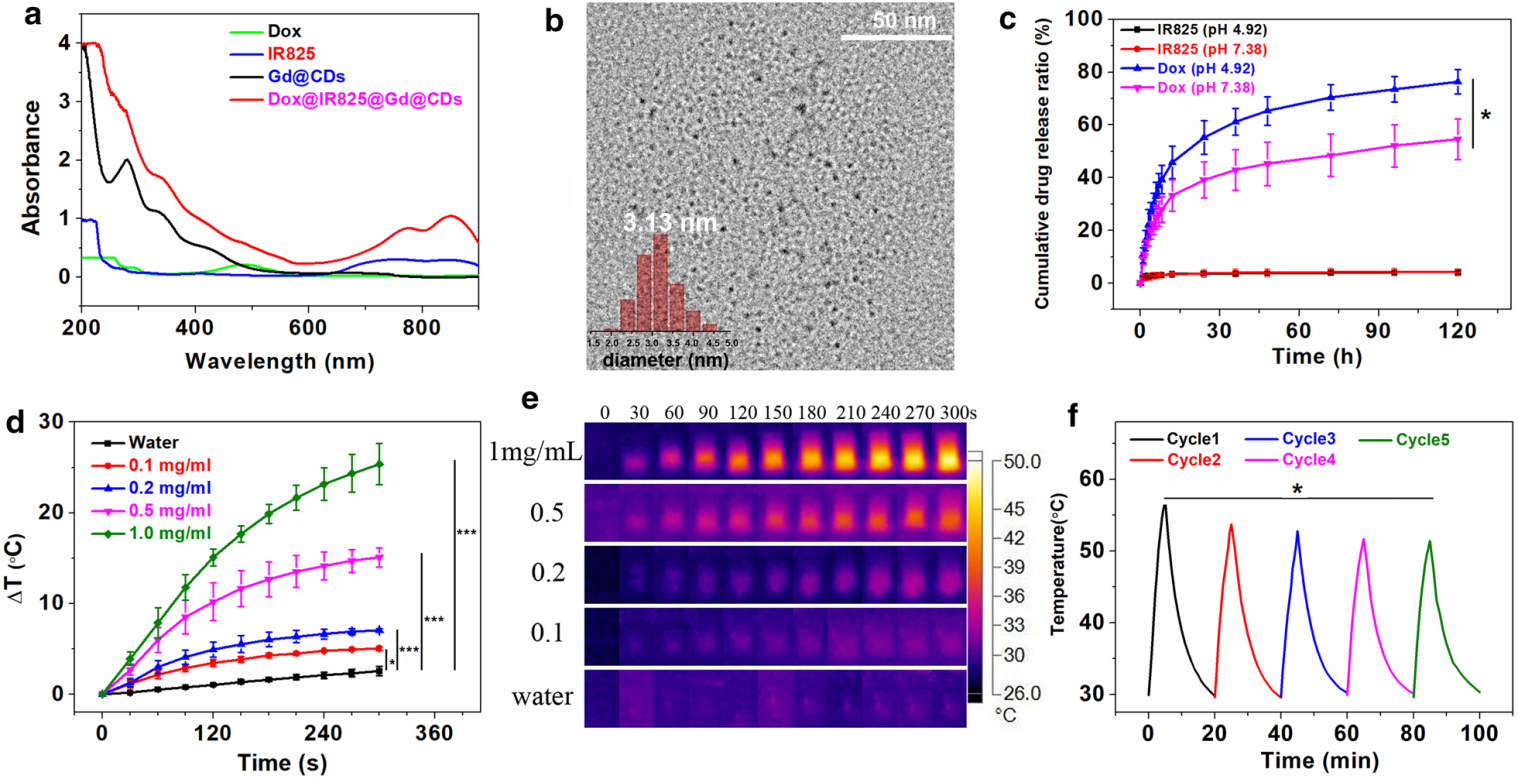
**a** The absorbance spectra of Gd@CDs before and after loading Dox and IR825; **b** HRTEM imaging of Dox@IR825@Gd@CDs (inset, statistical analysis by nanomeasurer software); **c** Dox and IR825 release curves at different pH (pH = 4.92, 7.38); **d** Temperature curves of deionized water and Dox@IR825@Gd@CDs solutions at different concentrations during NIR laser irradiation (808 nm, 3 W); **e** The IR thermal images of deionized water and Dox@IR825@Gd@CDs solution (1 mg/mL) after NIR laser irradiation (808 nm, 3 W); **f** Temperature increments of Dox@IR825@Gd@CDs (1 mg/mL) suspension under NIR laser irradiation (808 nm, 3 W) for five cycles (5 min for each cycle). Data expressed as mean ± SD, n = 3. *P < 0.05; **P < 0.01;***P < 0.005. *P* values represent the outcome of Multiple Comparisons in Analysis of Variance (ANOVA)

Photothermal agent IR825 and anti-tumor drug Dox were loaded on the surface of Gd@CDs. The as-synthesized Dox@IR825@Gd@CDs can be purified by filtration to remove free Dox and IR825. As shown Fig. 3a, a strong absorption peak in the range of 700–850 nm was observed, ascribing to the successful loading of IR825. The two typical peaks observed at 253 nm and 290 nm and the increased absorbance in the range of 470–550 nm proved that Dox had been loaded on the Gd@CDs. The loading efficiencies of Dox and IR825 were quantified to be 16.4% and 8.9% in Dox@IR825@Gd@CDs, respectively. Moreover, the drug encapsulation efficiencies of Dox and IR825 were quantified to be 68.8% and 83.3%, respectively. The zeta potentials of Dox and IR825 were 6.37 ± 2.5 mV and 3.68 ± 1.9 mV, while the surface charge of Gd@CDs was negative charge, a zeta potential was -16.7 ± 2.8 mV. Therefore, Dox and IR825 were absorbed on the carbon dots owing to electrostatic interactions. The FI-IR and XPS results strongly suggested that Gd@CDs were functionalized with various groups, such as hydroxyl groups, amide groups and carboxylic acid groups. Hence, the hydrogen bonds may be formed between Gd@CDs and drugs (Dox and IR825) as well as electrostatic interactions. The average diameter of Dox@IR825@Gd@CDs increased to 3.13 nm after loading Dox and IR825 (Fig. 3b) as a result of the increased amount of Dox and IR825. The stability of Dox@IR825@Gd@CDs in PBS with 10% FBS was investigated in vitro based on the DLS assays. The average hydrodynamic diameters were in the range from 228.8 ± 7.8 nm to 205.2 ± 9.8 nm, suggesting that the size was slightly decreased in 99 h, which showed that Dox@IR825@Gd@CDs were stable in physiological-mimicking environment (Additional file 1: Figure S8).

The release amount of IR825 reached 4.2% at pH 7.38 and 4.1% at pH 4.92 after 120 h (Fig. 3c). After statistical analysis, there was no difference between the release amount of IR825 at pH 7.38 and pH 4.92 after 120 h (*P* = 0.902). However, the release of Dox was significantly boosted on the acidic condition. 76.4% of Dox was released at pH 4.92, while only 55.6% of Dox was released at pH 7.38 (*P* = 0.014), which may be due to the protonation of amine groups on Dox under the acidic conditions. The release ratio of IR825 remained constant under different pH conditions, while Dox had a better release in the weak acidic environment. Considering the intracellular microenvironment of cancer cells was slightly acidic in the endosomal and lysosomal compartments [47, 48], such carbon nanoplatform showed a great potential for the delivery of anticancer drug Dox to cancer cells. Additionally, low release ratio of IR825 had a benefit to exert a photothermal effect on the tumor site.

In order to investigate the photothermal effect, various concentrations of Dox@IR825@Gd@CDs solutions were illuminated by 808 nm NIR laser at 3 W for 5 min (Fig. 3d, e). Nonobvious temperature fluctuations were observed in control (deionized water). By contrast, the rate of temperature elevation of Dox@IR825@Gd@CDs showed a typical concentration-dependent manner. The temperature of Dox@IR825@Gd@CDs at 0.1 mg/mL increased by 5.0 °C (*P* = 0.024), while that increased by 25.4 °C at 1.0 mg/mL after irradiation (*P* < 0.005), compared with the control. The thermal stability was a key factor to determine therapeutic efficacy of thermal therapy. Dox@IR825@Gd@CDs were irradiated with 808 nm laser for five on / off cycles to evaluate the light stability (Fig. 3f). The temperature of each cycles were recorded at 57, 53.7, 52.8, 51.7 and 51.4 °C. The temperature of the fifth cycle decreased 5.6 °C than that of the first cycle (*P* = 0.022). Slight decrease of the thermal effect would also be acceptable. Thus, Dox@IR825@Gd@CDs exhibited moderate photostability and could be implemented as a photothermal heater for photothermal therapy.

## Combined photothermal chemotherapy for TNBC in vitro

Cellular uptake assays. The 4T1 cells were incubated with Dox@IR825@Gd@CDs for 4 h, the characteristic red fluorescence of Dox mainly appeared in the cytosol of 4T1 cells, while the blue fluorescence of DAPI was showed in cell nuclei, which suggested that Dox@IR825@Gd@CDs easily entered 4T1 cells (Additional file 1: Figure S9).

**Fig. 4:**
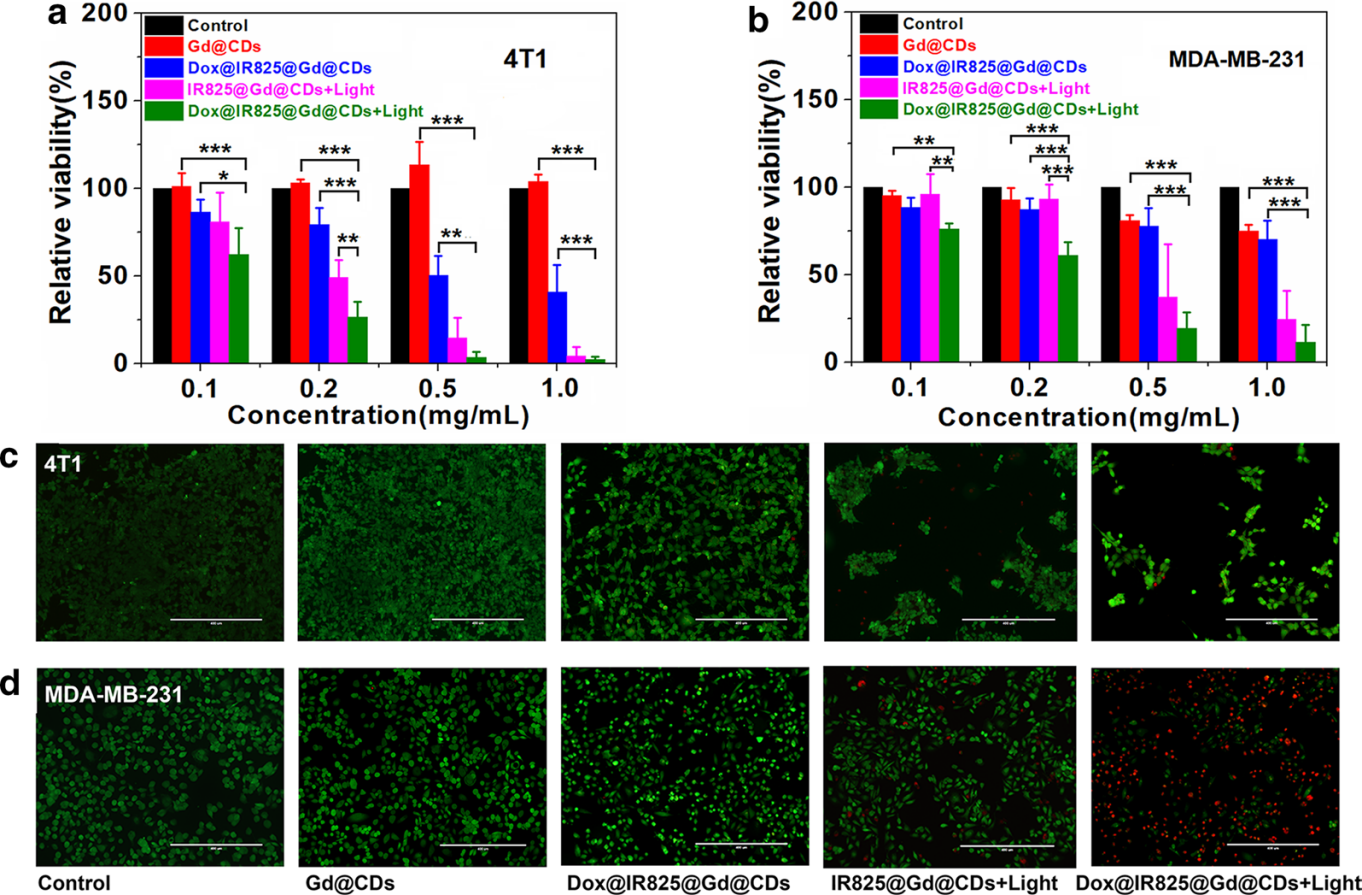
The cellular viability of 4T1 cells **a** and MDA-MB-231 cells **b** after various treatment; fluorescence images of the 4T1 cells **c** and MDA-MB-231 cells **d** stained with calcein-AM and PI after each treatment. Data expressed as mean ± SD, n = 3, *P < 0.05; **P < 0.01;***P < 0.005. *P* values represent the outcome of Multiple Comparisons in Analysis of Variance (ANOVA)

Viability assays. Encouraged by the above results, the TNBC cells killing efficiency of Dox@IR825@Gd@CDs was evaluated in vitro. The cytotoxicity of Gd@CDs was also investigated against 4T1 cells and MDA-MB-231 cells by CCK-8 assay (Fig. 4a, b). When the concentration of Gd@CDs increased to 1 mg/mL after 12 h incubation, 4T1 cells viability was still above 95%, while MDA-MB-231 cells viability was 75%. The results indicated the lower cytotoxicity and better bio-safety of Gd@CDs for anticancer therapy.

In vitro anticancer study revealed a concentration-dependent viability of Dox@IR825@Gd@CDs (Fig. 4a). For the treatment of 4T1 cells, it was shown that the chemotherapeutic efficacy of the Dox@IR825@Gd@CDs without irradiation was lower than single photothermal efficacy of IR825@Gd@CDs under NIR irradiation at 0.2 mg/mL (*P* = 0.002). The cellular viability of 4T1 with Dox@IR825@Gd@CDs under NIR irradiation (combined treatment groups) were decrease to 26%, compared with single photothermal group (*P* = 0.01). The time/pH-dependent drug release characteristics of Dox@IR825@Gd@CDs may lead to delayed therapeutic effects, thereby reducing cytotoxicity. Moreover, the anticancer efficacy of Dox@IR825@Gd@CDs drastically enhanced upon NIR irradiation for 5 min, and cancer cells were almost completely destroyed at high concentrations. When the concentration of Dox@IR825@Gd@CDs was 0.5 mg/mL, over 97% cells in the combined treatment groups were died compared to single chemotherapy (*P* = 0.003) or single phototherapy groups (*P* = 0.235). Note that hypoxia was often generated upon the growth of solid tumor, resulting in poor antitumor efficacy [49]. Photothermal effect could overcome the resistance caused by hypoxia [50]. Hence, Dox@IR825@Gd@CDs under irradiation greatly inhibited 4T1 cellular viability by combined photothermal chemotherapy. Interestingly, higher efficacy of Dox@IR825@Gd@CDs under irradiation was also observed in the treatment of MDA-MB-231 cells (Fig. 4b).

In order to visualize therapeutic efficiency combined photothermal chemotherapy treatment, the 4T1 cells and MDA-MB-231 cells were co-stained by calcein AM and PI to evaluate live and dead cells (Fig. 4c, d). 4T1 cells and MDA-MB-231 cells in the chemotherapeutic treatment groups (Dox@IR825@Gd@CDs without irradiation) had slightly decreased compared with the control groups. The live 4T1 cells and MDA-MB-231 cells treated with IR825@Gd@CDs under NIR irradiation had further decreased. Notably, 4T1 cells and MDA-MB-231 cells in the combinational therapy groups (Dox@IR825@Gd@CDs with NIR irradiation) showed a dramatic decrease in the number of live cells (green color). Taken together, these results demonstrated higher anticancer therapeutic efficiency of Dox@IR825@Gd@CDs when combined with PTT and chemotherapy.

### *MRI *in vitro* and *in vivo

**Fig. 5:**
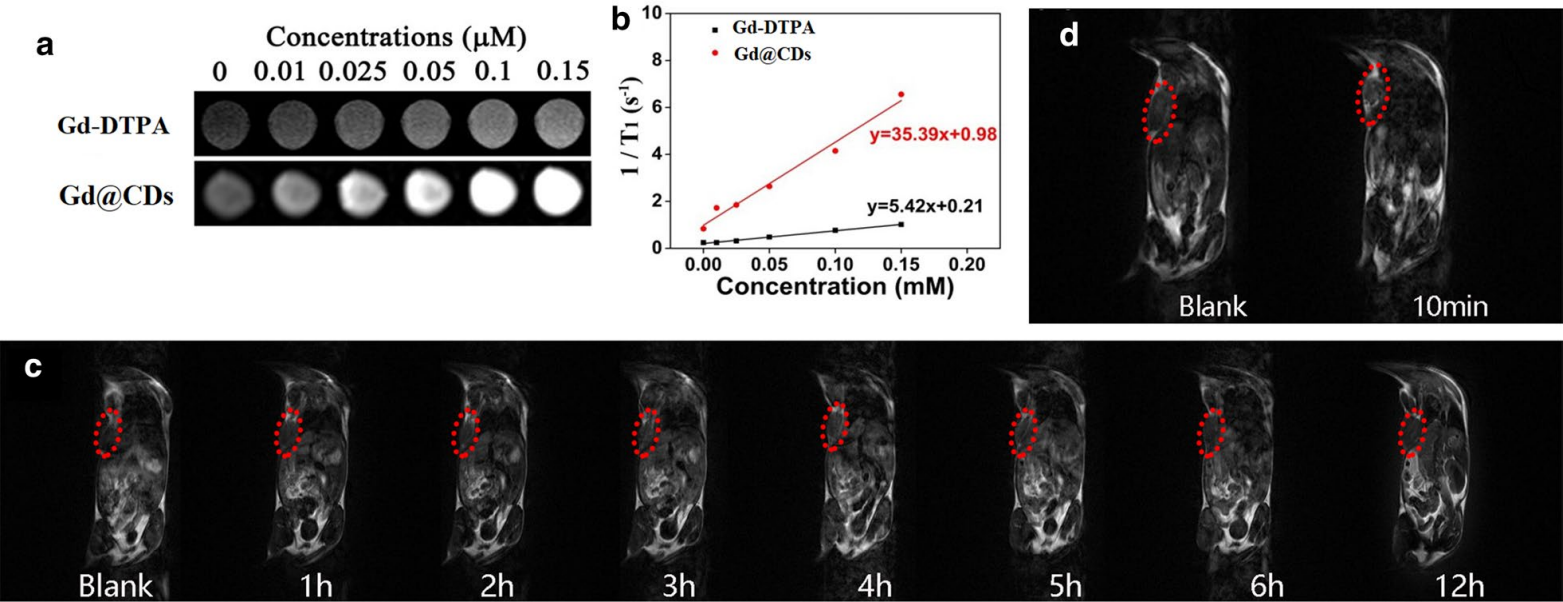
**a** In vitro MRI of Gd-DTPA and Gd@CDs; **b** Linear fit between Gd^3+^ concentration and relaxation rate; **c** In vivo MRI before and after intravenous injection Dox@IR825@Gd@CDs solution. **d** In vivo MRI of mice before and after intratumoral injection of Dox@IR825@Gd@CDs solution

Gd@CDs as T_1_ contrast agent improved the MRI effect by enhancing the longitudinal relaxation rate of surrounding water molecules. The brighter MRI was observed with increasing concentration of Gd@CDs (Fig. 5a). Under the same Gd dose, the MRI of Gd@CDs was brighter and clearer than that of Gd-DTPA, the commercial T_1_ MRI contrast agent, and the contrast effect was better than that of Gd-DTPA. According to Gd concentration and longitudinal relaxation rate R1 (1/T_1_), the linear equations of Gd@CDs and Gd-DTPA were obtained by fitting (Fig. 5b). Compared with the longitudinal relaxation rate R1 of Gd-DTPA, Gd@CDs showed a 6.53-fold higher R1. This may due to the coordination between Gd and phenolic hydroxyl groups, which prolonged the molecular rotation related time, improved the relaxation rate of Gd@CDs, and significantly increased the intensity of MRI contrast signals.

The Dox@IR825@Gd@CDs aggregation and distribution were determined by MRI in vivo. As shown in Fig. 5c, the MR signals of Dox@IR825@Gd@CDs at tumor regions increased gradually with time following intravenous injection, reaching a maximum level at 4 h-post-injection. Additionally, the MR signals of Dox@IR825@Gd@CDs were showed after 10 min post injection in situ (Fig. 5d), indicating that the MR images were much vaguer through intravenous injection than those via injection in situ. This may be due to small sizes of Dox@IR825@Gd@CDs, resulting facile and fast excretion. It was not an ideal approach by tail vein injection to enrich Gd dose in the tumor area. However, intratumoral injection was an easier and more time-saving strategy to endow tumors with the enrichment of Gd@CDs.

### *Combined photothermal chemotherapy for TNBC *in vivo

**Fig. 6:**
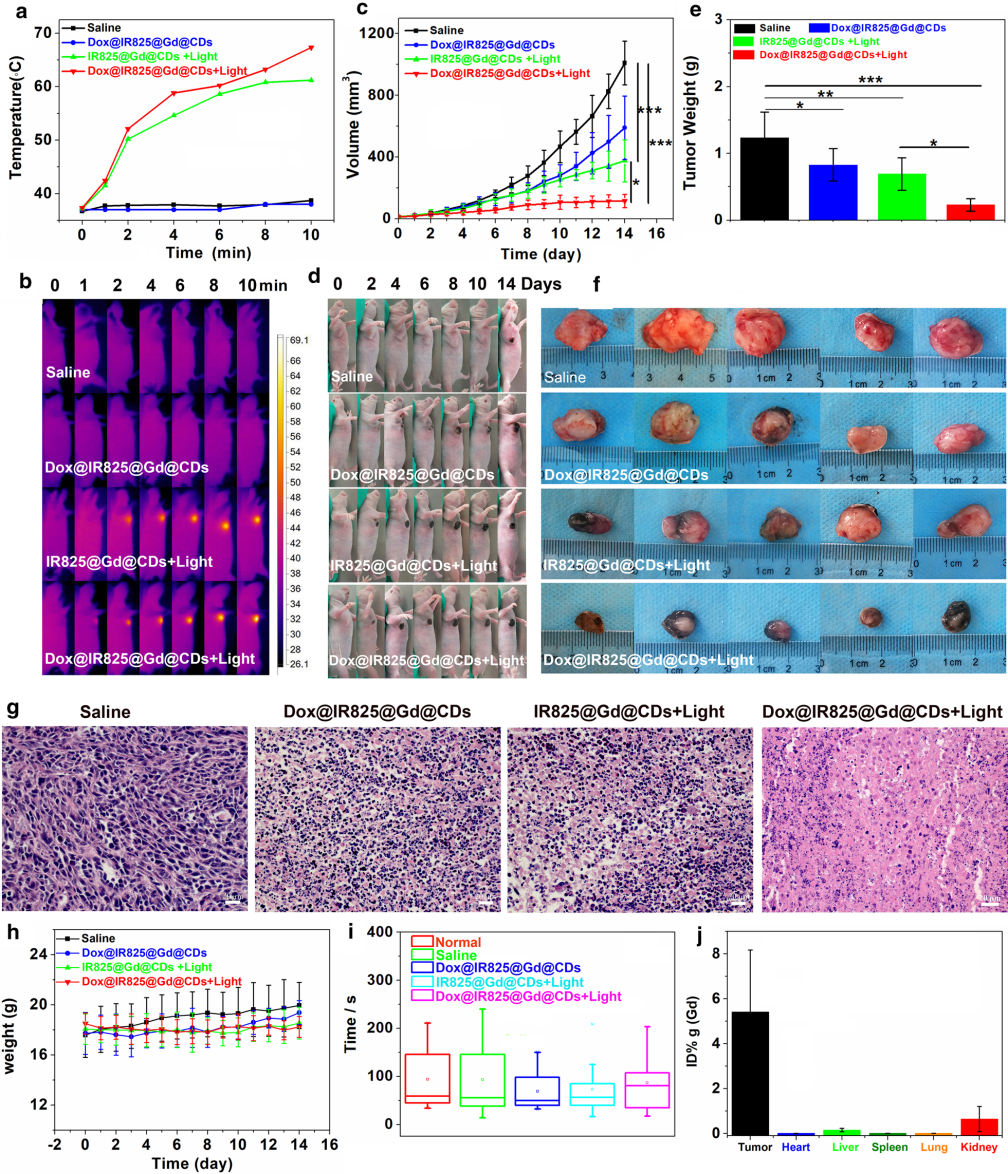
In vivo antitumor efficiency of different treatments: (1) saline (n = 5), (2) Dox@IR825@Gd@CDs (n = 5), (3) IR825@Gd@CDs + 808 nm irradiation (1.5 W, n = 5), (4) Dox@IR825@Gd@CDs + 808 nm irradiation (1.5 W, n = 5): **a** Temperature curves of 4T1 tumor-bearing mice after treatment. **b** Thermographic images of 4T1 tumor-bearing mice after treatment. **c** Tumor volume change curves. **d** Photographs of 4T1 tumor-bearing mice during different treatments. **e** Tumor weight collected from mice after 14 days treatments. **f** Photographs of tumor tissues collected from mice after 14 days treatments. **g** H&E-stained images of tumor slices. **h** Body weight changes. **i** Comparison of mice rotarod test performance between various treatments. **j** Drug biodistribution of 4T1 tumor-bearing mice following injection of Dox@IR825@Gd@CDs. *P < 0.05; **P < 0.01;***P < 0.005. *P* values represent the outcome of Multiple Comparisons in Analysis of Variance (ANOVA)

Inspired by the features of the Dox@IR825@Gd@CDs, we further evaluated combined therapeutic efficacy in vivo. Female nude mice bearing 4T1 tumors were chosen and randomly divided into four groups. The mice were intratumorally injected and the time-dependent temperature changes on the surface of tumor site were monitored using a thermal imager (Fig. 6a, b). The temperatures were rapidly increased under irradiation in IR825@Gd@CDs and Dox@IR825@Gd@CDs groups. The tumor tissue would suffer irreversible damage owe to the continued high temperature. The tumor volume of each mouse were measured every day during the treatment (Fig. 6c). As compared with the saline injected group, the tumors treated with chemotherapy alone (Dox@IR825@Gd@CDs without NIR irradiation) and photothermal therapy alone (IR825@Gd@CDs with NIR irradiation) grew slowly, but the tumor growth was only partially suppressed (Fig. 6c-f). By contrast, the mice treated with Dox@IR825@Gd@CDs under NIR irradiation exhibited the best tumor growth inhibition due to synergistic photothermal chemotherapeutic efficiency. The weights and sizes of isolated tumors after the combination therapy were the smallest in all the treated groups (Fig. 6e, f), showing excellent tumor inhibition efficacy of the combination therapy. In chemotherapy alone or photothermal therapy alone groups, tumor slices showed some tumor tissue necrosis (Fig. 6g). The shrinking and fragmented tumor cell nuclei were found and large numbers of granulocytes were dead. Moreover, tumor slices displayed that Dox@IR825@Gd@CDs under NIR irradiation killed more TNBC tumor cells thoroughly after synergistic photothermal chemotherapy (Fig. 6g). The body weight loss can be used as an indicator to evaluate the side effects of the treatment. Negligible differences in the body weights were observed in all treatment groups (*P* = 0.272, Fig. 6h). Mice rotarod test can be used for evaluation of murine motor coordination and the side effects of the treatment [51]. Based on mice rotarod test performance in various treated groups (Fig. 6i), there were no statistically significant difference for the average duration between the normal groups and experiment groups. Biodistribution of Dox@IR825@Gd@CDs in 4T1-tumor-bearing mice at 24 h post-inject was studied by measuring Gd levels in various organs using ICP-MS (Fig. 6j). Dox@IR825@Gd@CDs showed a rather high tumor accumulation at 5.4% of injected dose per gram tissue (ID/g), kidney accumulation at 0.6% ID/g. Furthermore, pathological morphological analysis was performed on the main organs of each treatment group to evaluate the systemic toxicity. Compared with the saline treatment groups, slices of the major organs showed no noticeable organ damages or inflammatory lesion (Additional file1: Figure S10). Thus, these results demonstrated that Dox@IR825@Gd@CDs with NIR irradiation efficiently inhibited tumor growth and enhanced the treatment efficacy after combined PPT and chemotherapy.

## Conclusions

In summary, Gd@CDs were synthesized through a facile hydrothermal method, that emitted blue fluorescence and exhibited excellent biocompatibility. Taking advantage of Gd@CDs, a noble theranostic nanoplatform (Dox@IR825@Gd@CDs) was developed that could be tailored to achieve loading of Dox and IR825, intracellular delivery, favorable MRI, excellent combination therapy with PTT and chemotherapy to enhance therapeutic effect against TNBC cells. Additionally, Dox@IR825@Gd@CDs presented low toxicity during the treatment, which provided a potential and singular approach to treat TNBC.

**Table 1.** Blood biochemistry analysis of mice after injection of saline (control group) or Gd@CDs (0.3 mg/kg and 3 mg/kg weight) for 16 days (x ± s)

biochemical markers	Control group	Gd@CDs 0.3 mg/kg	Gd@CDs 3 mg/kg	*P*
TP (g/L)	63.62 ± 4.91	64.48 ± 8.64	63.77 ± 3.08	0.96
ALT (U/L)	50.00 ± 12.55	56.58 ± 15.84	55.03 ± 15.95	0.73
AST (U/L)	230.42 ± 88.46	267.65 ± 75.51	244.10 ± 104.89	0.77
ALP (U/L)	193.48 ± 78.97	177.00 ± 44.67	178.70 ± 49.63	0.87
BUN (mmol/L)	10.46 ± 2.02	9.83 ± 1.56	8.62 ± 2.75	0.35
TC (mmol/L)	2.32 ± 0.56	2.14 ± 0.56	2.32 ± 0.34	0.78
TG (mmol/L)	1.54 ± 0.69	1.60 ± 0.50	1.25 ± 0.27	0.47

*TP* Total protein, *ALT* alanine aminotransferase, *AST* aspartate aminotransferase, *ALP* alkaline phosphatase, *BUN* blood urea nitrogen, *TC* total cholesterol, *TG* triglyceride

## Supplementary Information

**Additional file 1.** The experimental section of radical scavenging activity and hemolysis assay. **Figure S1.** Hydrodynamic diameter and fluorescence lifetime of Gd@CDs, and fluorescence intensity of Gd@CDs at different temperatures. **Figure S2.** The Influencing factors of fluorescence properties on Gd@CDs. **Figure S3.** Photobleaching characteristics and evaluation of free radical scavenging activity. **Figure S4.** Hemolysis experiment, and cellular viability of Gd@CDs with 293 T cells. **Figure S5.** H&E stained sections of major organs at 24 h post injection of Gd@CDs. **Figure S6.** Body weight changes of mice during 16 days treatments. **Figure S7**. H&E stained sections of major organs after 16 days treatments. **Figure S8**. Stability of Dox@IR825@Gd@CDs. **Figure S9**. The cellular uptake of Dox@IR825@Gd@CDs by 4T1 cells. **Figure S10**. H&E stained sections of major organs of 4T1 tumor-bearing mice after 14 days treatments.

## Data Availability

The data are available in the main manuscript, supplementary Information files, and from the corresponding authors upon reasonable request.

## Abbreviations

TNBC: Triple negative breast cancer
Gd: Gadolinium
Gd@CDs: Gd-encapsulated carbon dots
Dox: Doxorubicin hydrochloride
NIR: Near-infrared
MRI: Magnetic resonance imaging
Gd-DTPA: Gadopentetate dimeglumine
Gd-DOTA: Gadolinium 1,4,7,10-tetraazacyclododecane-1,4,7,10-tetraacetic acid
NSF: Nephrogenic systemic fibrosis
CDs: Carbon dots
PTT: Photothermal therapy
DHCA: 3,4-Dihydroxyhydrocinnamic acid
EDA: 2,2′-(Ethylenedioxy)bis(ethylamine)
GdCl_3_: Anhydrous gadolinium chloride
DPPH: 1,1-Diphenyl-2-picrylhydrazyl
FBS: Fetal bovine serum
CCK-8: Cell Counting Kit-8
NMR: Nuclear magnetic resonance
FT-IR: Fourier transform infrared
ICP-MS: Inductively coupled plasma mass
XPS: X-ray photoelectron spectroscopy
ANOVA: Analysis of variance
DLS: Dynamic light scattering
TP: Total protein
ALT: Alanine aminotransferase
AST: Aspartate aminotransferase
ALP: Alkaline phosphatase
BUN: Blood urea nitrogen
TC: Total cholesterol
TG: Triglyceride
