# Supplementary material for: NIR-laser-triggered gadolinium-doped carbon dots for magnetic resonance imaging, drug delivery and combined photothermal chemotherapy for triple negative breast cancer
Source: J Nanobiotechnology. 2021 Mar 2;19:64. doi: 10.1186/s12951-021-00811-w (PMC7923633; doi:10.1186/s12951-021-00811-w)
Supplement: Supplementary file 1 — Additional file 1. The experimental section of radical scavenging activity and hemolysis assay. Figure S1. Hydrodynamic diameter and fluorescence lifetime of Gd@CDs, and fluorescence intensity of Gd@CDs at different temperatures. Figure S2. The Influencing factors of fluorescence properties on Gd@CDs. Figure S3. Photobleaching characteristics and evaluation of free radical scavenging activity. Figure S4. Hemolysis experiment, and cellular viability of Gd@CDs with 293 T cells. Figure S5. H&E stained sections of major organs at 24 h post injection of Gd@CDs. Figure S6. Body weight changes of mice during 16 days treatments. Figure S7. H&E stained sections of major organs after 16 days treatments. Figure S8. Stability of Dox@IR825@Gd@CDs. Figure S9. The cellular uptake of Dox@IR825@Gd@CDs by 4T1 cells. Figure S10. H&E stained sections of major organs of 4T1 tumor-bearing mice after 14 days treatments. [file 12951_2021_811_MOESM1_ESM.docx]

**Additional file 1**

**NIR-Laser-Triggered Gadolinium-doped Carbon Dots for Magnetic Resonance Imaging, Drug Delivery and Combined Photothermal chemotherapy for Triple Negative Breast Cancer**

Qunjiao Jiang ^1†^, Li Liu ^1†^, Qiuying Li ^1^, Yi Cao ^3^, Dong Chen ^2^, Qishi Du ^2^, Xiaobo Yang ^1^, Dongping Huang ^1^*, Renjun Pei ^3^*, Xing Chen ^1^*, and Gang Huang ^2^*

*Correspondence: [dongpinghuang@gxmu.edu.cn](mailto:dongpinghuang@gxmu.edu.cn;); [rjpei2011@sinano.ac.cn](mailto:peij2010@sinano.ac.cn); [chenx63@163.com](mailto:chenx63@163.com;); [wangyi.07@163.com](mailto:wangyi.07@163.com;)

† These authors contributed equally to this work.

1. School of Public Health, Guangxi Medical University, Nanning 530000, China

2. State Key Laboratory of Non-food Biomass and Enzyme Technology, Guangxi Academy of Sciences, Nanning 530007, China

3. Key Laboratory of Nano-Bio Interface, Suzhou Institute of Nano-Tech and Nano-Bionics, Chinese Academy of Sciences, Suzhou 215123, China

**Radical scavenging activity**

Various concentrations of Gd@CDs solutions were incubated with DPPH (50 μM) methanol solution. After 1.5 h incubation in the dark, the absorbance was recorded at 517 nm. The inhibition was calculated according to equation (1):

$\mathrm{Inhibition}\left( \% \right)=\frac{A_{c}-A_{s}}{A_{c}}\times100$ (1)

*A_c_* and *A_s_* were the absorbance of the solution in absence and presence of Gd@CDs. EC_50_ was the half maximal effective concentration, corresponding to the 50% inhibition of radicals.

**Hemolysis assay**

Fresh human red blood cells (HRBCs) were collected *via* centrifugation at 1500 rpm for 10 min before washing five times with PBS buffer (pH = 7.4). Packed HRBCs were dispersed in PBS solution (4 mL). Diluted HRBCs solution (200 μL) and Gd@CDs solution (the final concentration: 0.02, 0.04, 0.06, 0.08, 0.10 mg/mL) were incubated at 37 °C for 3 h while deionized water and PBS buffer were used as positive and negative controls. After centrifugation at 1500 rpm for 10 min, the absorbance at 540 nm of the supernatant solution was determined. Hemolytic rate was calculated according to equations (2):

$Hemolytic rate\left( \% \right)=\frac{A_{s}-A_{n}}{A_{p}-A_{n}}\times100$% (2)

*A_s_, A_p_, A_n_* were the absorbance of the sample, positive and negative controls.


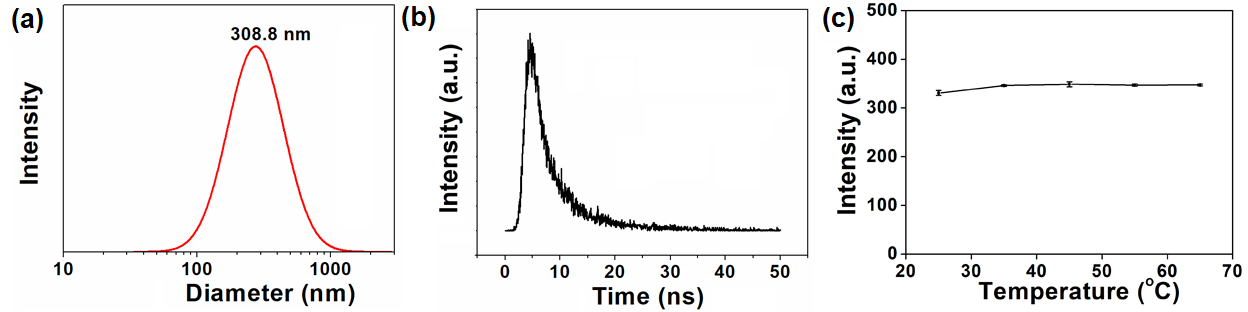


Figure S1 (a) Hydrodynamic diameter of Gd@CDs measured by DLS, (b) The fluorescence lifetime of Gd@CDs, (c) The fluorescence intensity of Gd@CDs at different temperatures.


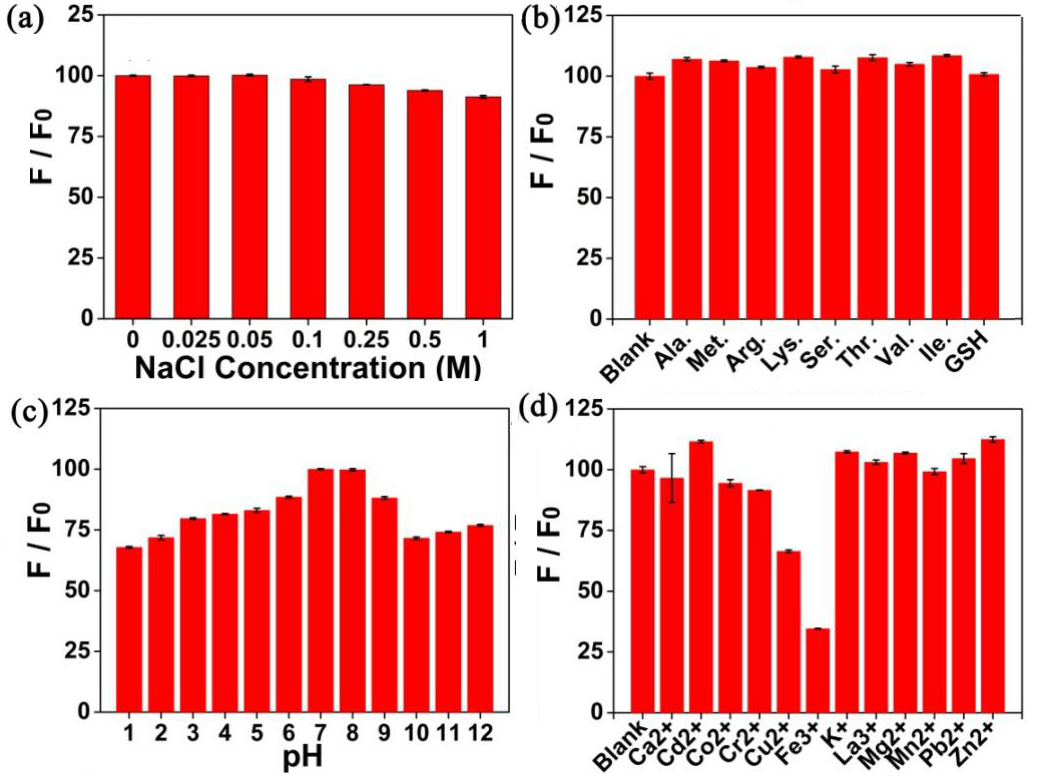


Figure S2. The influencing factors on fluorescence properties of Gd@CDs: (a) NaCl, (b) amino acid, (c) pH, (d) Metal ions. Data expressed as mean ± SD, n = 3.


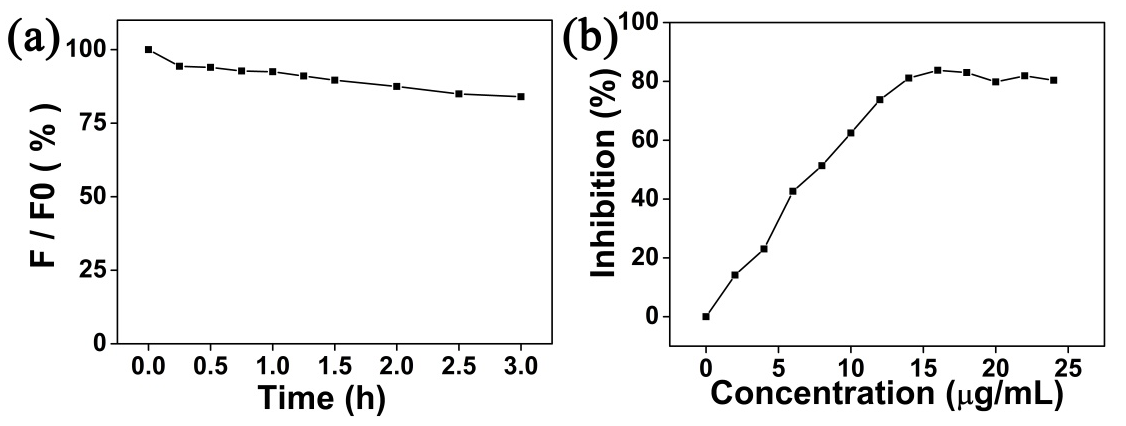


Figure S3. (a) Photobleaching characteristics after Xe lamp irradiation, (b) Evaluation of free radical scavenging activity of Gd@CDs.


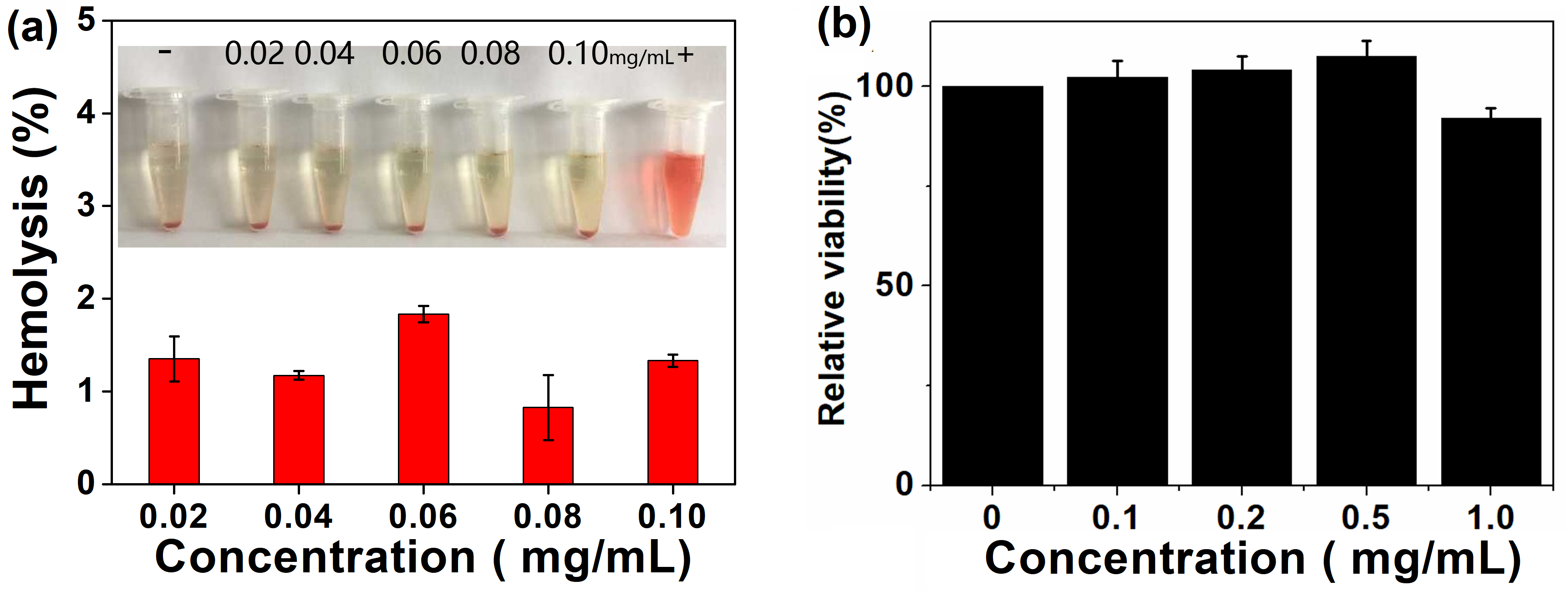


Figure S4. (a) Hemolysis experiment of Gd@CDs, (b) The cellular viability of 293T cells in the presence of Gd@CDs solution. Data expressed as mean ± SD, n = 3.


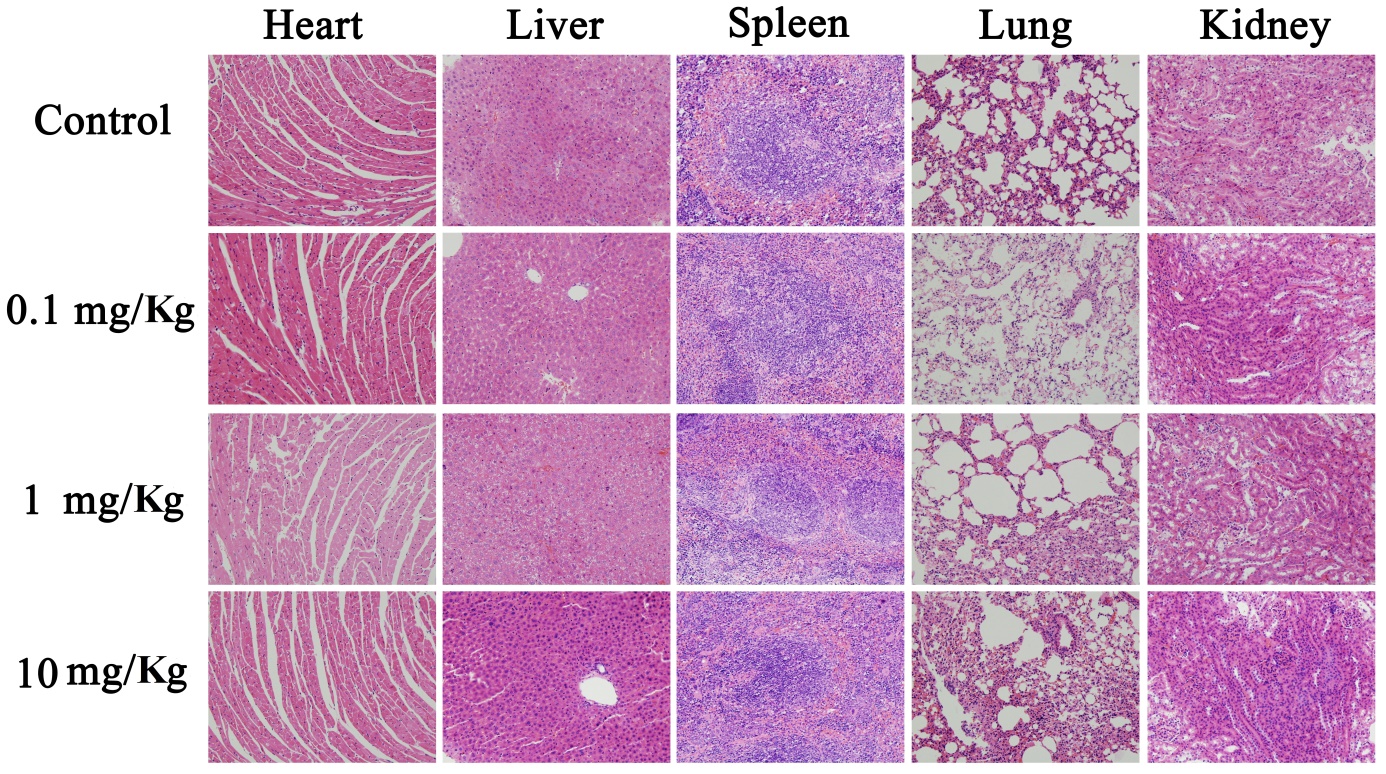


Figure S5. H&E stained sections of heart, liver, spleen, lung and kidney organs at 24 h post injection of Gd@CDs solution.


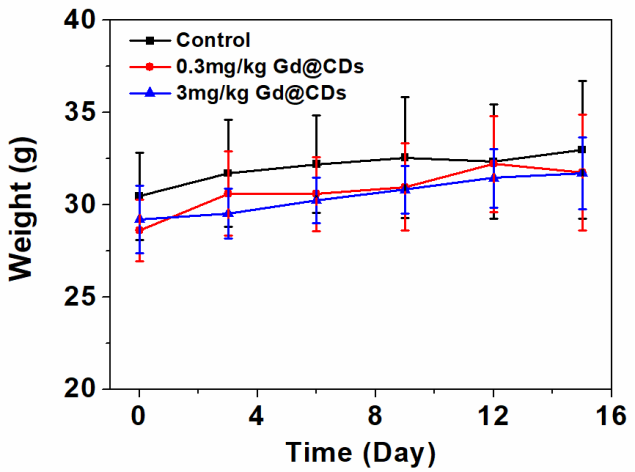


Figure S6. Body weight changes of mice in each group (Control: saline; 0.3 mg/Kg Gd@CDs solution; 3 mg/Kg Gd@CDs solution) during 16 days treatments. There was no statistical difference between the control and treated groups. Data expressed as mean ± SD, n = 6.


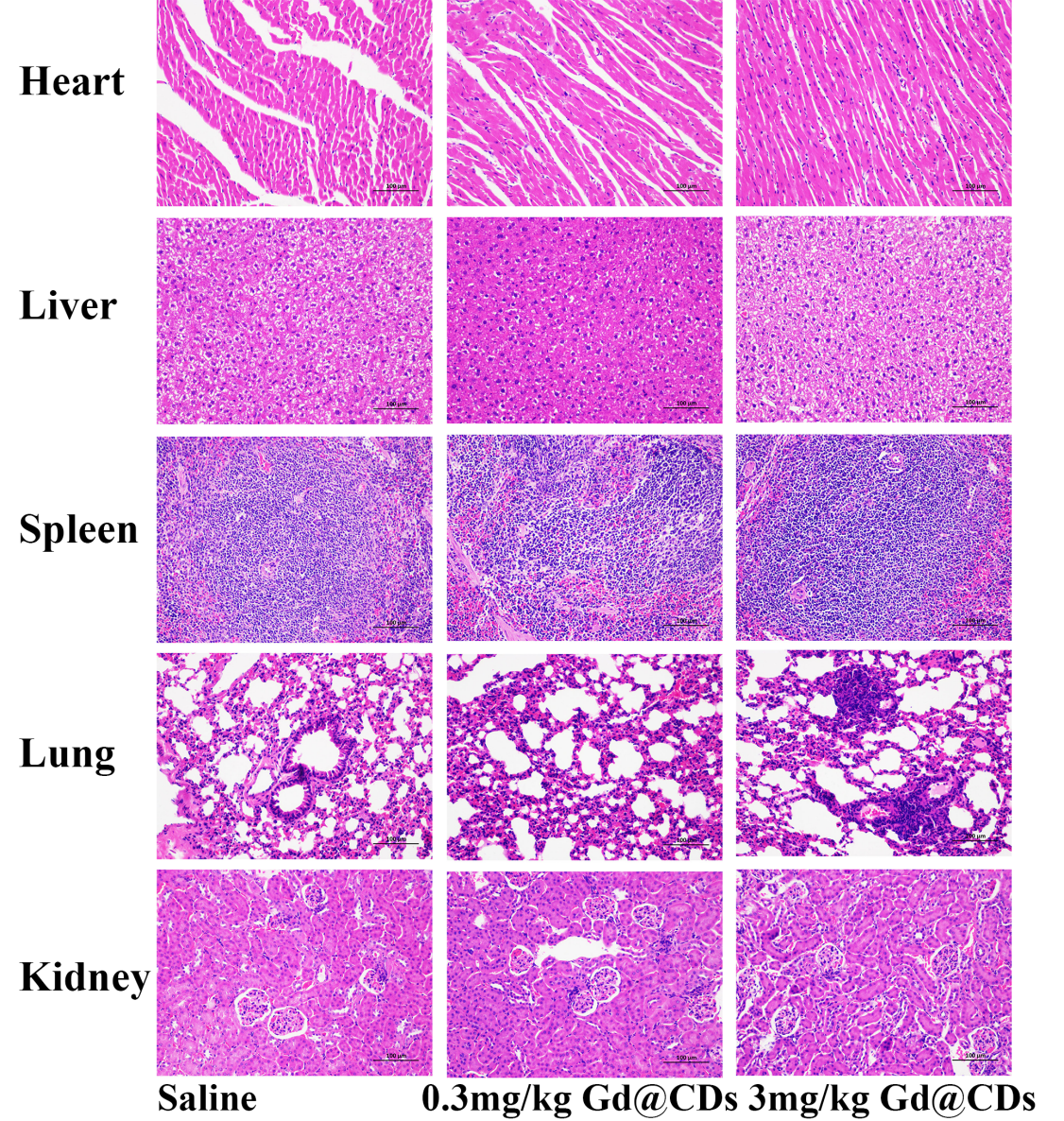


Figure S7. H&E stained sections of heart, liver, spleen, lung and kidney organs at 16 days post injection.


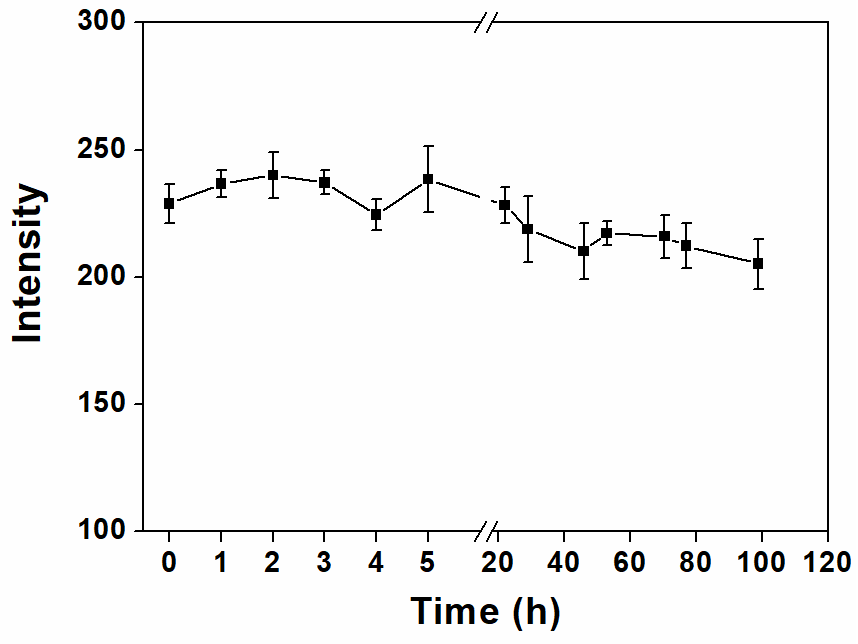


Figure S8. Stability of Dox@IR825@Gd@CDs in PBS with 10 vol% FBS during 99 h. Data expressed as mean ± SD, n = 3.


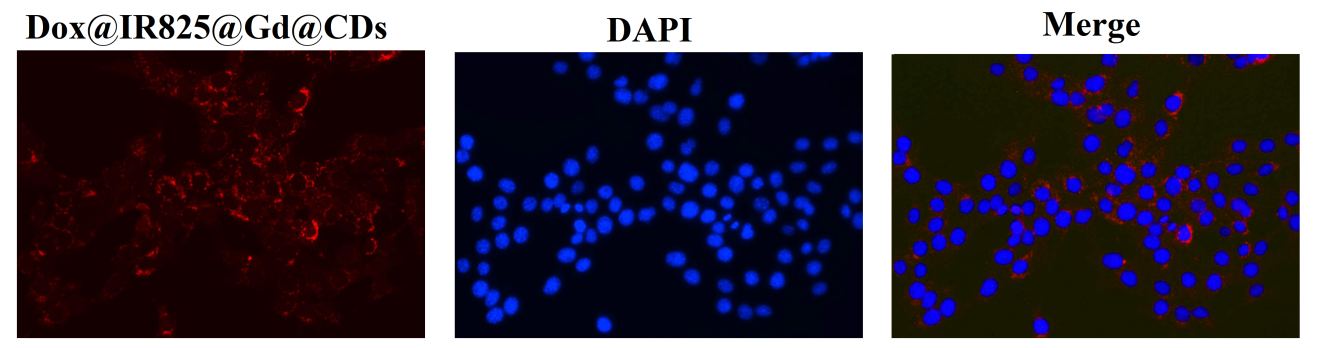


Figure S9. The cellular uptake of Dox@IR825@Gd@CDs by 4T1 cells.


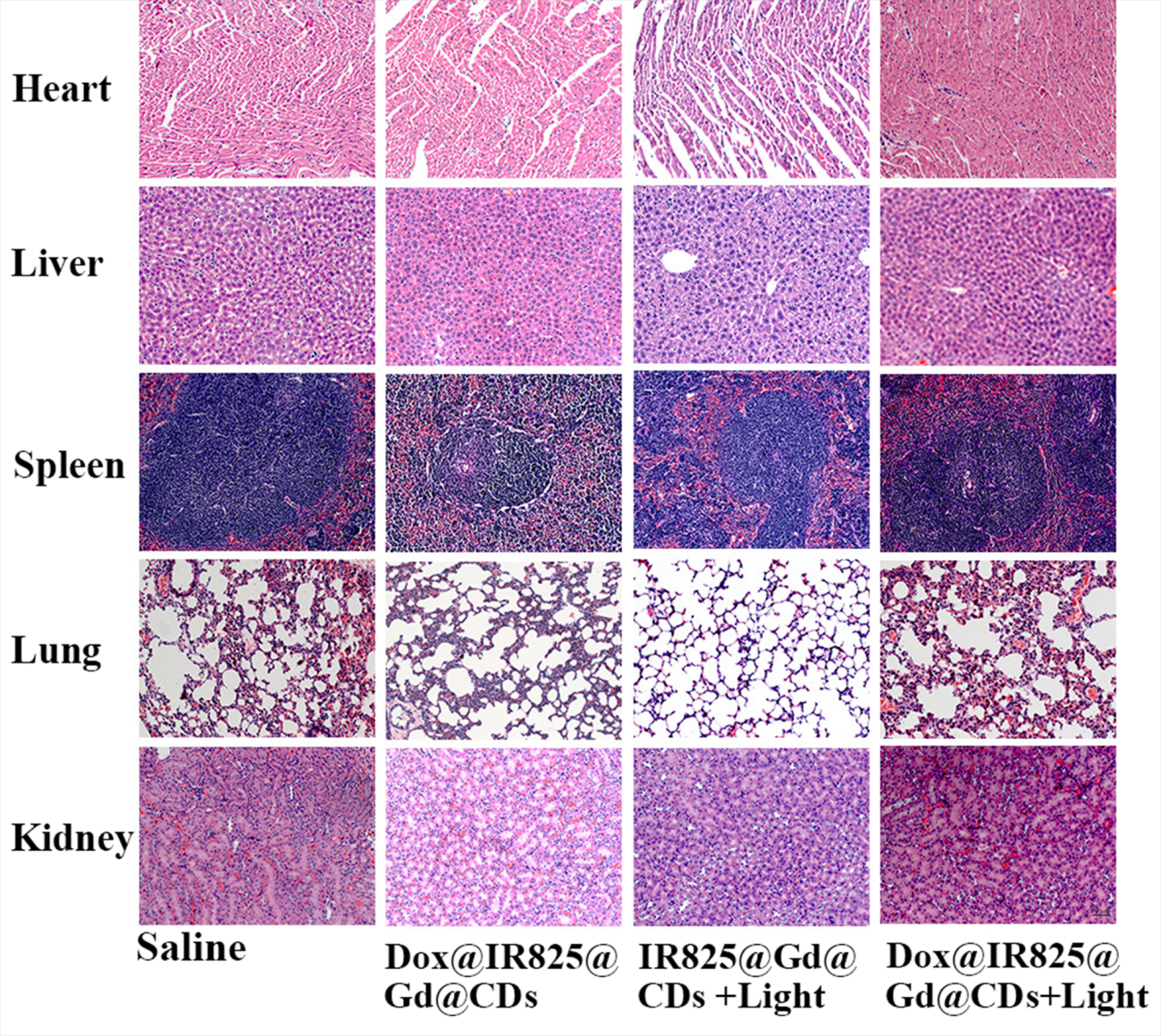


Figure S10. H&E stained sections of heart, liver, spleen, lung and kidney organs of 4T1 tumor-bearing mice after 14 Days treatments.
